# Correction of Faulty Sensors in Phased Array Radars Using Symmetrical Sensor Failure Technique and Cultural Algorithm with Differential Evolution

**DOI:** 10.1155/2014/852539

**Published:** 2014-01-29

**Authors:** S. U. Khan, I. M. Qureshi, F. Zaman, B. Shoaib, A. Naveed, A. Basit

**Affiliations:** ^1^School of Engineering & Applied Sciences, ISRA University, Islamabad 44000, Pakistan; ^2^Electrical Department, Air University, Islamabad 44000, Pakistan; ^3^Institute of Signals, Systems and Soft Computing (ISSS), Islamabad 44000, Pakistan; ^4^Electronic Engineering Department, IIU, H-10, Islamabad 44000, Pakistan

## Abstract

Three issues regarding sensor failure at any position in the antenna array are discussed. We assume that sensor position is known. The issues include raise in sidelobe levels, displacement of nulls from their original positions, and diminishing of null depth. The required null depth is achieved by making the weight of symmetrical complement sensor passive. A hybrid method based on memetic computing algorithm is proposed. The hybrid method combines the cultural algorithm with differential evolution (CADE) which is used for the reduction of sidelobe levels and placement of nulls at their original positions. Fitness function is used to minimize the error between the desired and estimated beam patterns along with null constraints. Simulation results for various scenarios have been given to exhibit the validity and performance of the proposed algorithm.

## 1. Introduction

In adaptive beamforming, null steering and beam steering are hot research areas. It has direct application in radar, sonar, and mobile communication [1–3]. In the literature various analytical and computational methods are available to concentrate on the issue of null steering [4–6]. The condition becomes more demanding and complicated when a sensor fails in the active antenna array. The excitation of these sensors is to accomplish desired radiation pattern. In case of sensor failure, the sidelobe level (SLL) raise and nulls are displaced, which is highly unwanted. It is very expensive in terms of time and budget to replace the defective sensor regularly. Hence the weights of active sensors in the same array should be recalculated and readjusted to create a new pattern close to the original one. Recently few algorithms have been proposed to correct the damaged pattern of the array [7–10].

In the last few decades Radar technology has developed very rapidly. The radar commonly used nowadays is known as phased array radar. In this radar the whole input array transmits the same signal with different delay and a beam is formed towards the area of interest [11]. The advantages of beam include the electronic steering instead of mechanical steering and a high processing gain at the transmitter. The phased array radar used the phase shifting in the input waveform to steer a beam electronically in the direction of the target instead of mechanical steering. Array design is one of the most active research area in phased array radars in which the sensors are arranged together to form an array. The phase shifters adjust the phase in such a way that a beam is formed in the desired direction. The width of the beam depends on the number of sensors in the array. By increasing the number of sensors in an array, the beam becomes sharper and thus more efficient in detecting the targets with smaller size. Now if one or more sensors become damaged, the radars cannot detect the target correctly. Researchers are still working to recalculate and adjust the weights of the active array to get the pattern near the original one. By recalculating the weights of the faulty array, the radar will improve its capabilities in such a way that the radar can perform searching, tracking, and weapon guidance at the same time.

The evolutionary computing technique has been doing well in solving numerous problems in search and optimization due to the impartial nature of their operations which can still be present in situations with no domain knowledge. The search method used by evolutionary algorithms (EAs) is impartial having no domain knowledge to guide the search method. Domain knowledge serves as a method to reduce the search space by pruning unnecessary parts of the solution space and by promoting required parts. Cultural algorithm is based on the principle to bias the search method with prior knowledge about the domain, as well as the knowledge during the evolution method.

Among EC techniques, differential evolution (DE) is considered to be one of the powerful and reliable tools to optimize the problems in any engineering field [12–14]. The DE is a technique based on stochastic searches, in which function parameters are programmed as floating-point variables. The DE algorithm has a simple structure, convergence speed, flexibility, and robustness, with only some parameters required to be put by a user. The application of CAs in DE is a different strategy to get the performance and local search better. The previous work on null steering in failed antenna arrays is presented in [15]. The technique tries to restore the previous nulls pattern by using particle swarm optimization (PSO). All the above EC based techniques have discussed the SLL reduction and null steering in failed array, but no one is solving the issue of null depth and null steering at their original positions using CADE for the correction of faulty arrays. Authors in their previous work [16] have used the symmetrical element failure technique to achieve the required null depth level and first null beamwidth and [17] for fault finding in failed array antenna. Memetic computing algorithms are stochastic population based methods that have been established to be dominant and forceful to solve optimization problems. The advantages of cultural algorithm (CA) with evolutionary algorithms (EAs) include global search capability and consistent performance in any field of engineering and technology [18–20].

In this paper, the proposed algorithm developed three issues in case of sensor failure. These are raised in sidelobe levels, displacement of nulls from their original positions, and diminishing of null depth. We propose a symmetrical sensor failure (SSF) method that provides better results in terms of null depth. Moreover, the SSF method has deeper first null which is another big improvement over single sensor failure. The first null depth in beamforming is of great importance. To address the other two issues, we have used a cultural algorithm with differential evolution (CADE) to reduce the sidelobe levels and positions of nulls reverse to their original positions by adjusting the current weights of active sensors. A hybrid method based on the memetic computing algorithm is proposed, which combines the cultural algorithm with differential evolution (CADE) for the reduction of sidelobes and placement of nulls. Different simulation results are provided to confirm the performance of the proposed approach. The rest of the paper is organized as follows. The problem formulation is discussed in Section 2, while in Section 3 the proposed solution is provided.  Section 4 describes the simulations and the results while Section 5 concludes the paper and proposes some future work.

## 2. Problem Formulation

Consider a linear array of 17 sensors in which all the sensors are placed symmetrically about the origin. The total number of sensors is *N* = 2*M* + 1. The array factor in this healthy set up with uniformly spaced sensors, nonuniform weight, and progressive phase excitation will be [21]

(1) $$\begin{array}{c} \text{AF}\left(\theta_{i}\right)=\overset{M}{\underset{n=-M}{\sum }}w_{n}\exp\left(jn\left(kd\cos\theta_{i}+\beta \right)\right), \end{array}$$

where *w*_*n*_ is the nonuniform weight of *n*th sensor whereas *n* = 0, ±1, ±2,…, ±*M*. The spacing between the adjacent sensors is *d*, while *θ* is the angle from broadside. *k* = 2*π*/*λ* is the wave number with *λ* as wavelength. The progressive phase shift *β* = −*kd*cos⁡⁡*θ*_*s*_ and *θ*_*s*_ is steering angle for the main beam. The damage array factor for 7th sensor failure is given by the expression below:

(2) $$\begin{array}{c} \text{AF}\left(\theta_{i}\right)=\overset{M}{\underset{\begin{array}{c} n=-M\\ n\neq 7 \end{array}}{\sum }}w_{n}\exp\left(jn\left(kd\cos\theta_{i}+\beta \right)\right). \end{array}$$

The nonuniform weights of 17 sensors with 7th symmetry sensor failures are as follows:

(3) [w−8,w−7,w−6,w−5,w−4,…,w−1,w0, w1,…,w4,w5,w6,w7,w8].

It is assumed that the *w*_7_ sensor fails in the antenna array given in (3). One can clearly monitor from Figure 1 that due to single sensor failure the radiation pattern is damaged in terms of sidelobe levels, null depth, and displacement of the nulls from their original position. So, the goal of this job is to recover the null depth, sidelobe levels, and null steering at their original positions. Different methods are available in the literature to correct the damage pattern of sensor failures; however, none of them is able to achieve the required null depth level.

## 3. Proposed Solution

In this section, we develop the proposed solution based on SSF. As we had assumed the damage of *w*_7_ sensor, we lost the null depth as given in Figure 1. For SSF method we also force the sensor *w*_−7_ to be zero as shown in (3). From Figure 2, it is clear that symmetric sensor failure maintains the null depth almost as close to that of the original array. The damage array factor for 7th symmetrical sensor failure is given by

(4) $$\begin{array}{c} \text{AF}\left(\theta_{i}\right)=\overset{M}{\underset{\begin{array}{c} n=-M\\ n\neq \pm 7 \end{array}}{\sum }}w_{n}\exp\left(jn\left(kd\cos\theta_{i}+\beta \right)\right). \end{array}$$

Although we have achieved better null depth level due to SSF, but the sidelobe levels and positioning of nulls are still a problem to be taken into account, for which we will use the cultural algorithm with differential evolution (CADE) for the reduction of sidelobes and placement of nulls in the required positions.

**Pseudocode 1:**
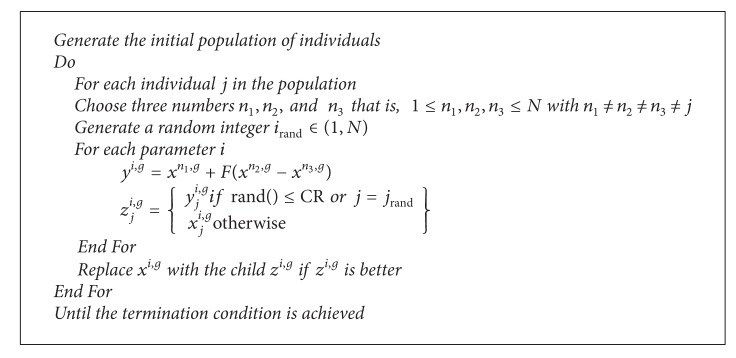
The pseudocode of the differential evolution algorithm.

**Pseudocode 2:**
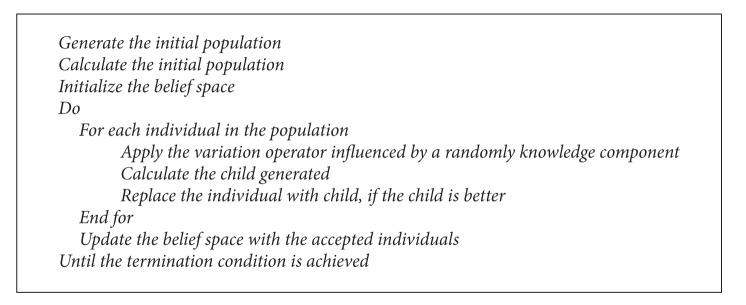
The pseudocode of the cultural algorithm.

### 3.1. Differential Evolution (DE)

DE is an EA and was developed by Storn and Price which is used to solve real valued optimization problems [22]. The DE is a method based on stochastic searches. The DE algorithm presents easy structure, convergence speed, flexibility, and robustness, with only some parameters required to be set by a user. However, this faster convergence of DE results in a higher possibility of searching near a local optimum or getting early convergence. Differential evolution is based on a mutation operator, which adds an amount obtained by the difference of two randomly chosen individuals of the present population. The problem to be solved has *N* decision variables; *F* and *CR* are parameters given by the user and given in Table 1. Computing the difference between two individuals which are selected randomly from the population, in fact the algorithm estimating the gradient in that region and this technique is a proficient way to self-adapt the mutation operator. The pseudocode for the DE is given in Pseudocode 1.

### 3.2. The Cultural Algorithm with Differential Evolution (CADE)

Differential evolution is used as a population space in the cultural algorithm. CAs have been developed to model the evolution of the cultural component of an evolutionary computational system over time as it accumulates experience. As a result, CAs can provide a clear method for global knowledge and a framework within which to model self-adaptation in an evolutionary system.

Cultural algorithms consist of three components, a population space, belief space, and a communication protocol as shown in Figure 3. First one is population space that contains the population to be evolved and the mechanisms for its estimate. The population space consists of a set of possible solutions to the problem; in our problem, the population space is DE. Second, one is a belief space that represents the bias that has been acquired by the population during its problem solving process. In CAs, the information acquired by a member of the population can be shared with the entire population. The third one is the communication protocol that is used to determine the interface between the population and the beliefs.

CAs model has two levels of evolution. One is the population level and the other is belief space level. In addition to a population space, CA has a belief space in which the beliefs acquired from the population's evolution can be stored and integrated. An acceptance function is used to generate beliefs by gleaning the experience of individuals from the population space. In return, this belief space can bias the evolution of the population component by means of the influence function. The belief space itself also evolves by the adjust function [23]. In the present work, the belief space is divided into two knowledge components.

#### 3.2.1. Situational Knowledge

Situational knowledge stores individuals from population space which provides the direction for other individuals. Situational knowledge consists of the best example *P* found along the evolutionary process. It represents a leader for the other individuals to follow. The variation operators of differential evolution are influenced in the following way

(5) $$\begin{array}{c} y^{i,g}=P_{i}+F\left(x^{n_{2},g}-x^{n_{3},g}\right), \end{array}$$

where *P*_*i*_ is the *i*th component of the individual stored in the situational knowledge. This way, we use the leader instead of a randomly chosen individual for the recombination, getting the children closer to the best point found. The update of the situational knowledge is done by replacing the stored individual *P*, by the best individual found in the current population, *x*_best_ only if *x*_best_ is better than *P*.

#### 3.2.2. Normative Knowledge

The normative knowledge contains the intervals for the decision variables where good solutions have been found, in order to move new solutions towards those intervals. The normative knowledge includes a scaling factor, *ds*_1_ to influence the mutation operator adopted in differential evolution. The following expression shows the influence of the normative knowledge of the variation operators

(6) $$\begin{array}{c} z_{j}^{i,g}=\{\begin{array}{cc} x^{n_{1},g}+F\left(x^{n_{2},g}-x^{n_{3},g}\right) & \text{if}\;\;x^{n_{1},g}<l_{i}\\ x^{n_{1},g}-F\left(x^{n_{2},g}-x^{n_{3},g}\right) & \text{if}\;\;x^{n_{1},g}>u_{i}\\ x^{n_{1},g}+\left(\frac{u_{i}-l_{i}}{ds_{i}}\right)*F\left(x^{n_{2},g}-x^{n_{3},g}\right) & \text{otherwise}, \end{array} \end{array}$$

where *l*_*i*_ and *u*_*i*_ are the lower and upper bounds, respectively, for the *i*th decision variable. The update of the normative knowledge can reduce or expand the intervals stored on it. An extension takes place when the accepted individuals do not fit in the current interval, while a reduction occurs when all the accepted individuals lie in the current interval, and the extreme values have an improved fit and are feasible. The values *ds*_*i*_ are updated with the difference (*x* ^*n*_2_,*g*^ − *x* ^*n*_3_,*g*^) found of the variation operators of the prior generation. Normative knowledge leads individuals to jump into the good range if they are not already there. The normative knowledge is updated as follows, let us consider *x*_*a*1_, *x*_*a*2_, *x*_*a*3_,…, *x*_*an*_accepted__ be the accepted individuals in the current generation and *x*_min_*i*__ and *x*_max_*i*__ belong to (*a*1, *a*2, *a*3,…, *n*_accepted_) and the accepted individuals with minimum and maximum values for the parameter *i*:

(7) $$\begin{array}{c} u_{i}=\{\begin{array}{cc} x_{i,\text{ma}{\text{x}}_{i}} & \text{if}\;\;x_{i,\text{ma}{\text{x}}_{i}}>u_{i}\;\;\text{or}\;\;f\left(x_{\text{ma}{\text{x}}_{i}}\right)<U_{i}\\ u_{i} & \text{otherwise}, \end{array}\\ l_{i}=\{\begin{array}{cc} x_{i,\text{mi}{\text{n}}_{i}} & \text{if}\;\;x_{i,\text{mi}{\text{n}}_{i}}<l_{i}\;\;\text{or}\;\;f\left(x_{\text{mi}{\text{n}}_{i}}\right)<L_{i}\\ l_{i} & \text{otherwise}. \end{array} \end{array}$$

If *l*_*i*_ and *u*_*i*_ are updated, the values of *L*_*i*_ and *U*_*i*_ will be done in the same way. The *ds*_*i*_ are updated with the greatest difference of |*x*_*i*,*r*1_ − *x*_*i*,*r*2_| found during the variation operators at the prior generation.

The flow chart and pseudocode for CADE is shown in Pseudocode 2 and Figure 3.

### 3.3. Null Constraint (NC)

A jamming signal located at a particular angle wants to be eliminated, in case of satellite, radar, and mobile communication applications. For a uninformed array, to put a null at a particular angle *θ*_*i*_, we want [24]

(8) $$\begin{array}{c} \text{AF}\left(\theta_{i}\right)=w^{H}s\left(\theta_{i}\right)=0, \end{array}$$

where

(9) $$\begin{array}{c} s\left(\theta_{i}\right)={\left[\begin{array}{c} \exp\left(-j\left(\frac{N-1}{2}\right)kd\cos\theta_{i}\right)\\ \exp\left(-j\left(\frac{N-3}{2}\right)kd\cos\theta_{i}\right)\\ \vdots \\ \exp\left(j\left(\frac{N-3}{2}\right)kd\cos\theta_{i}\right)\\ \exp\left(j\left(\frac{N-1}{2}\right)kd\cos\theta_{i}\right) \end{array}\right]}_{N\times 1} \end{array}$$

and **w** ^*H*^ is *N* × 1 vector which is defined as

(10) $$\begin{array}{c} w={\left[w_{-M},w_{-M+1},\ldots ,w_{0},\ldots ,w_{M-1},w_{M}\right]}^{T}. \end{array}$$

The null constraint is given as

(11) $$\begin{array}{c} w^{H}s\left(\theta_{i}\right)=0,\;i=1,2,\ldots ,M_{0}. \end{array}$$

We may define an *N* × *M*_0_ constraint matrix **C** as

(12) $$\begin{array}{c} C=\left[s\left(\theta_{1}\right),s\left(\theta_{2}\right),\ldots ,s\left(\theta_{M_{0}}\right)\right], \end{array}$$

where *θ*_*i*_ for *i* = 1,2,…, *M*_0_ is the direction of null. Our goal is to optimize the squared weighting error subject to the condition that

(13) $$\begin{array}{c} w^{H}C=0. \end{array}$$

Our constraint is that the columns of **C** should be orthogonal to the weight vector **w**. Accordingly we may define *G*_*i*_, *i* = 1,2 and *G* as follows:

(14) $$\begin{array}{c} G_{1}=\overset{P}{\underset{i=1}{\sum }}{\left[\left|\text{A}{\text{F}}_{d}(\theta_{i})-\text{A}{\text{F}}_{\text{CADE}}(\theta_{i})\right|\right]}^{2}, \end{array}$$

(15) $$\begin{array}{c} G_{2}={||w^{H}C||}^{2}, \end{array}$$

(16) $$\begin{array}{c} G=G_{1}+G_{2}. \end{array}$$

Hence *G* is the fitness function for the problem given above which are to be minimized. Best chromosome will give the minimal value of *G*. The first term in (16) is used for SLL reduction, where AF_*d*_(*θ*_*i*_) represents the desired pattern and AF_CADE_(*θ*_*i*_) is the pattern obtained by using CADE. The second term in (16) is used for jammer suppression and placement of nulls at their original positions after sensor failure.

## 4. Simulation Results

In the simulation, a classical Dolph-Chebyshev linear array of 17 sensors with *λ*/2 intersensor spacing is used as the test antenna. The array factor in this case represents a −35 dB constant SLL with the nulls at specific angles. Analytical techniques are used to find out the nonuniform weights for classical Dolph-Chebyshev array. In case of sensor failure, cultural algorithm with differential evolution (CADE) is used for the reduction of sidelobes and placement of nulls in the required positions.

*Case a*. At the first instant the *w*_7_ sensor is assumed to fail. After sensor failure the radiation pattern is destroyed, which results in an increase of the SLL and displacement of null positions. In order to regain the symmetry, its mirror sensor weight *w*_−7_ is forced to zero. We achieve the desired null depth level (NDL) and deeper first null depth level (FNDL) as compared to that of nonsymmetric case. The SLL rises to −32.32 dB due to the *w*_7_ sensor failure, while due to SSF of the *w*_7_ sensor, the SLL is −26.53 dB. The advantage of SSF is deeper nulls, especially, the first null. The SLL and FNDL for a damage array of single sensor failure and SSF are shown in Table 2. It is clear from Figure 2 that SSF maintains better FNDL as compared to that of single sensor failure.

After optimization by a cultural algorithm with differential evolution (CADE), the SLL of the *w*_7_ sensor failure are reduced to −32.99 dB while due to SSF, the SLL is reduced to −28.35 dB. The recovery of one null due to 7th sensor failure and SSF to its original position *θ*_1_ = 19.93° is shown in Figures 4 and 5. The comparison of recovered pattern with 7th sensor and SSF for one null imposed is given in Table 3. The recovered NDL of SSF is seven dB deeper than that of 7th sensor failure.

Figures 6 and 7 show the recovery of two nulls at angles of *θ*_1_ = 19.93° and *θ*_2_ = 34.88°, respectively, for 7th sensor failure and SSF. The SLL and NDL for the corresponding nulls are given in Table 4. The NDL of the SSF is deeper as compared to the 7th sensor failure.

Now we check the recovery of three nulls at the required positions. Figures 8 and 9 show the recovery of three nulls originally at angles of *θ*_1_ = 19.93°, *θ*_2_ = 34.88°, and *θ*_3_ = 45.44° for 7th sensor failure and SSF. The SLL and NDL for the corresponding nulls are given in Table 5. The NDL of all nulls in SSF is deeper than that of 7th sensor failure.

Now the recovery of five nulls for 7th sensor failure and SSF originally at positions 19.93°, 34.88°, 45.44°,62.02°, and 68.94° is carried out and shown in Figures 10 and 11. A comparison of SLL and NDL for the recovery of five nulls is given in Table 6. In each case SSF produces deeper nulls compared to the 7th sensor failure. From simulation it is observed that we have received deeper depth of nulls in SSF scenarios as compared to 7th sensor failure discussed above.

*Case b*. In this case, we discuss the failure of *w*_4_ sensor. If the sensor *w*_4_ fails due to any reason, the whole radiation pattern became damage. After optimization, the SLL reduces and nulls are steered back to their original positions as shown in Figure 12. Then to create symmetry we also force *w*_−4_ equal to zero, to achieve the required null depth level. The advantage of symmetry sensor failure is to get deeper null depth level. The number of nulls achieved in 7th symmetry sensor failure is six, and in case of 4th symmetry sensor failure the number of nulls received are three. From the simulation results, it is clear that the number of nulls reduces by one as the sensors get damage near the centre sensor. After optimization by CADE, the SLL reduces and nulls are steered back to their previous positions at angles *θ*_1_ = 34.89°, *θ*_2_ = 54.31°, and *θ*_3_ = 68.9° as shown in Figure 13. The null depth level for single and SEF is given in Table 7.

*Case c*. In this section, we discuss the possibility of getting failure near the centre sensor, if the sensor *w*_1_ fails due to unforeseen reason. From Figure 14 it is clear that its SLL increases and nulls are damaged and also lose null depth. To create the symmetry, we also force its mirror sensor weight *w*_−1_ equal to zero. The one advantage of symmetry sensor failure is to get the deeper null depth level and, on the other hand, due to *w*_1_ SSF its beamwidth also decreases. In case of 7th symmetry sensor failure, the nulls are six, and in 4th SSF the achievable nulls are three but we received only one null in case of *w*_1_ SSF as shown in Figure 15. The null depth level for single and SEF is given in Table 8.

*Case d*. The main beam can be steered at any desired angle. If the user changes their position than the main beam can be steered in the desired direction.  Figure 16 shows the corrected pattern with recovered nulls at main beam pointing at *θ*_*s*_ = 110°. The main beam can be steered in the direction of the desired user at any particular angle. The array factor for 2*M* + 1 sensors in terms of main beam direction *θ*_*s*_ is given by

(17) $$\begin{array}{c} \text{AF}\left(\theta_{i}\right)=\overset{M}{\underset{n=-M}{\sum }}w_{n}\exp jnkd\left(\cos\theta_{i}-\cos\theta_{s}\right), \end{array}$$

where *θ*_*s*_ is the main beam direction to which it can be steered to the desired angles.

## 5. Conclusion and Future Work

We have proposed symmetric sensor failure (SSF) technique along cultural algorithm with differential evolution for the correction of faulty arrays. The null depth of all nulls, especially the first one, has been achieved with the help of SSF technique. Null steering at their original positions and sidelobe reduction has been achieved by a cultural algorithm with differential evolution and using a proper fitness function demanding the sidelobe reduction and null constraints. Due to 7th SSF the number of nulls is six and in 4th SSF the achievable nulls are three but in case of *w*_1_ SSF we received only one null with reduced beamwidth. The simulation result shows that as the faulty sensor gets near the central sensor the number of nulls reduces by one. The reduction in the corrected sidelobe level comes at the cost of broader main beam. The corrected pattern has beamwidth broader than that of the original one. Using the approach of symmetric sensor failure, with the reduction of the SLL, we can steer single, double, and multiple nulls in the direction of known interferences. This method can be extended to planar arrays.

## Figures and Tables

**Figure 1:**
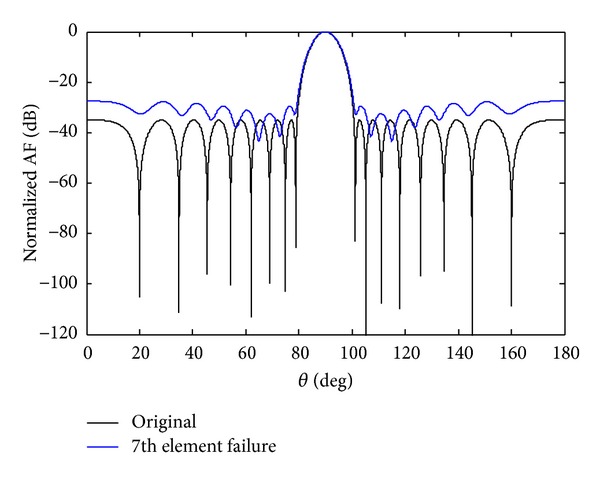
The original Chebyshev array and the *w*_7_ sensor damage pattern.

**Figure 2:**
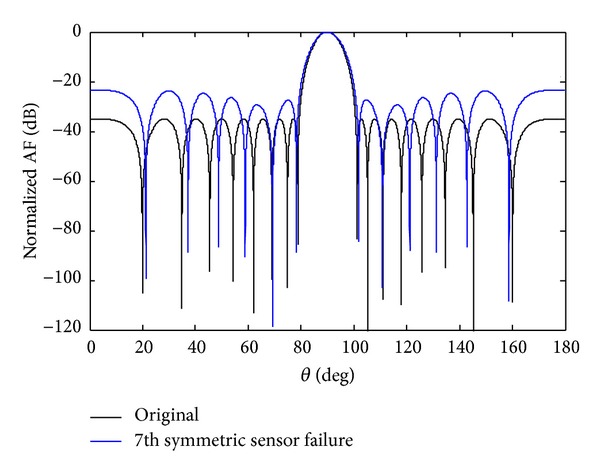
The original Chebyshev array and the *w*_7_ symmetric sensor failure.

**Figure 3:**
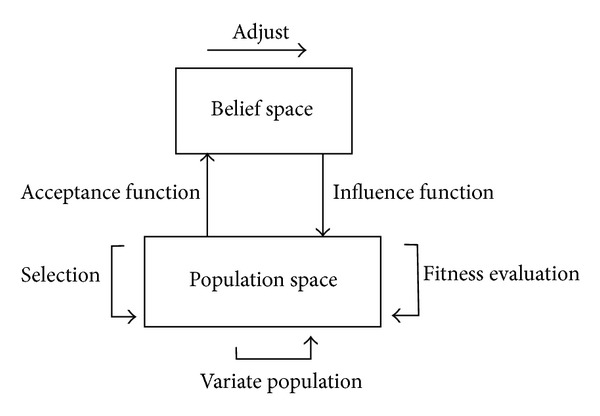
The flow diagram of the cultural algorithm.

**Figure 4:**
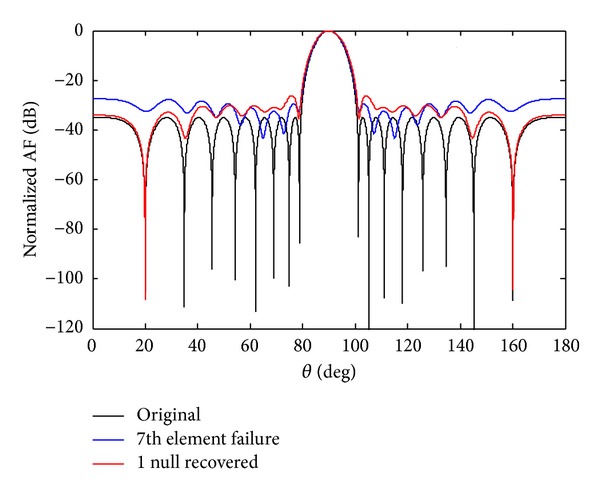
The original radiation pattern, the *w*_7_ sensor damage, and recovery of one null.

**Figure 5:**
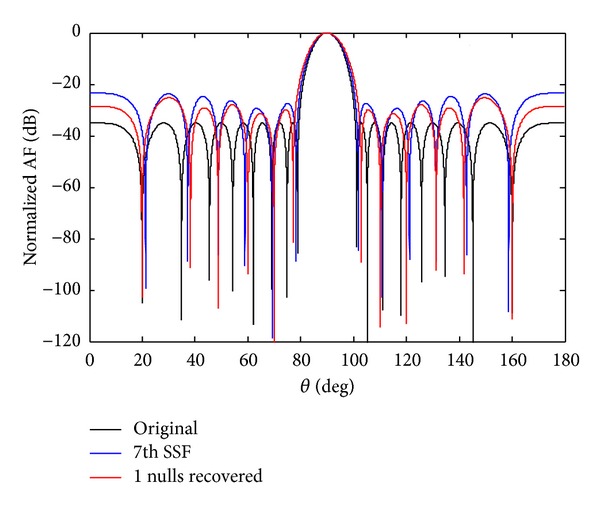
The original radiation pattern, the *w*_7_ SSF, and recovery of one null.

**Figure 6:**
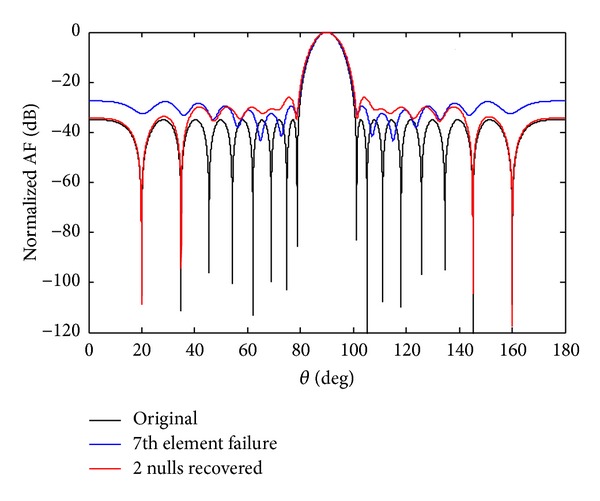
The original radiation pattern, the *w*_7_ sensor damage, and recovery of two nulls.

**Figure 7:**
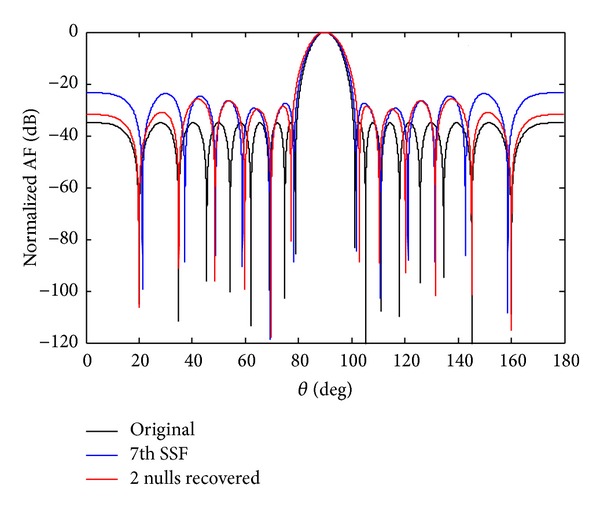
The original radiation pattern, the *w*_7_ SSF, and recovery of two nulls.

**Figure 8:**
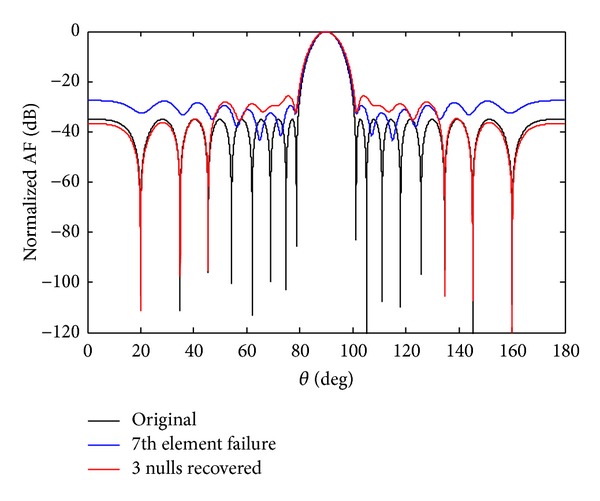
The original radiation pattern, the *w*_7_ sensor damage, and recovery of three nulls.

**Figure 9:**
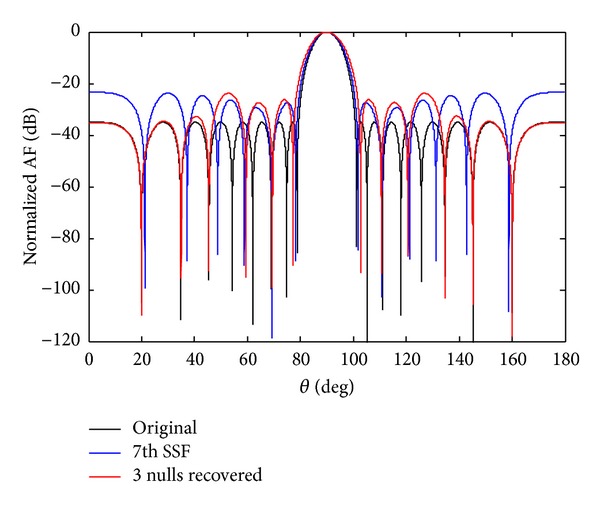
The original radiation pattern, the *w*_7_ SSF, and recovery of three nulls.

**Figure 10:**
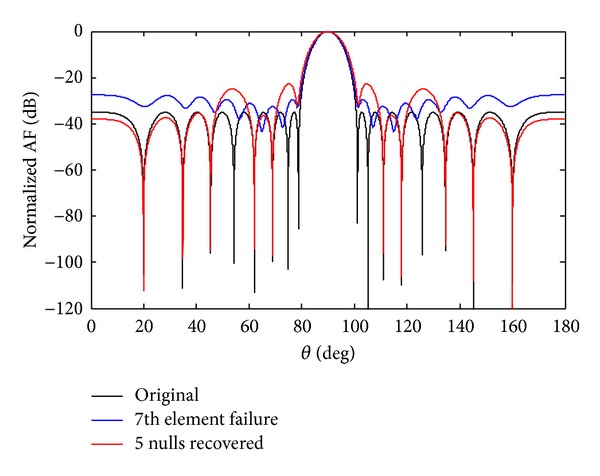
The original radiation pattern, the *w*_7_ sensor damage, and recovery of five nulls.

**Figure 11:**
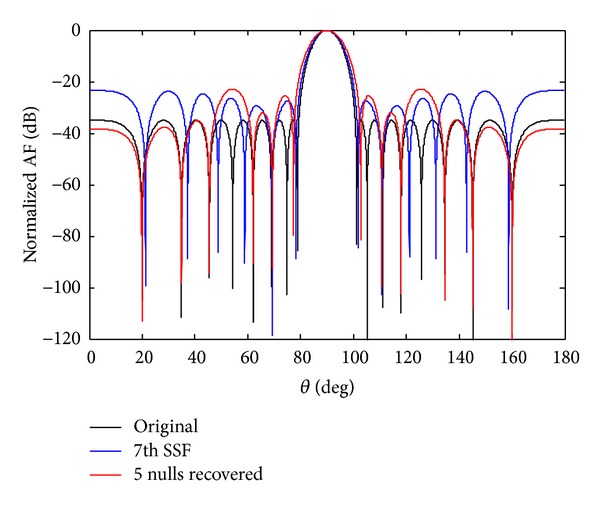
The original radiation pattern, the *w*_7_ SSF, and recovery of five nulls.

**Figure 12:**
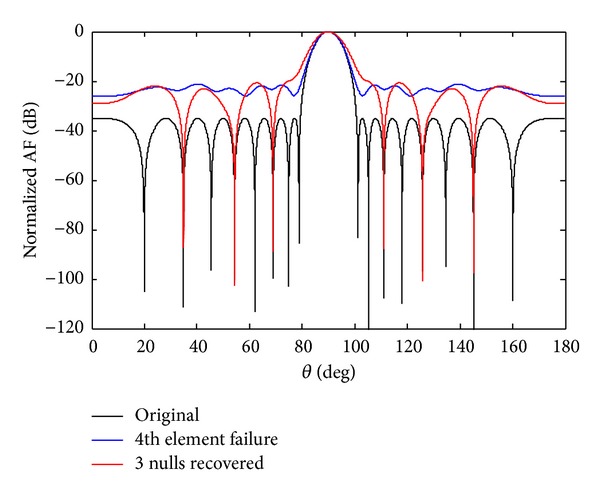
The original radiation pattern, the *w*_4_ sensor failure, and recovery of three nulls.

**Figure 13:**
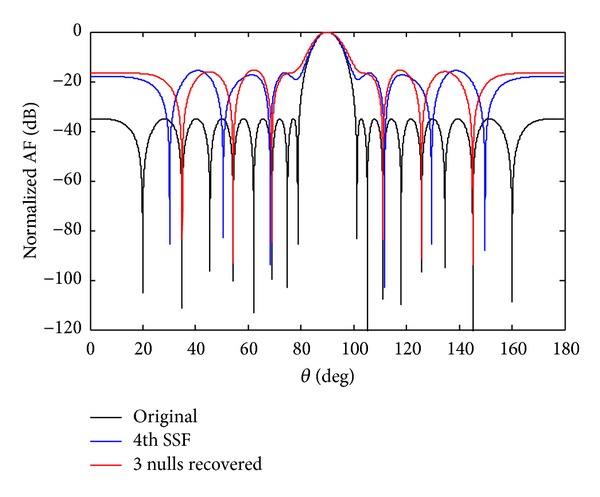
The original radiation pattern, the *w*_4_ SSF, and recovery of three nulls.

**Figure 14:**
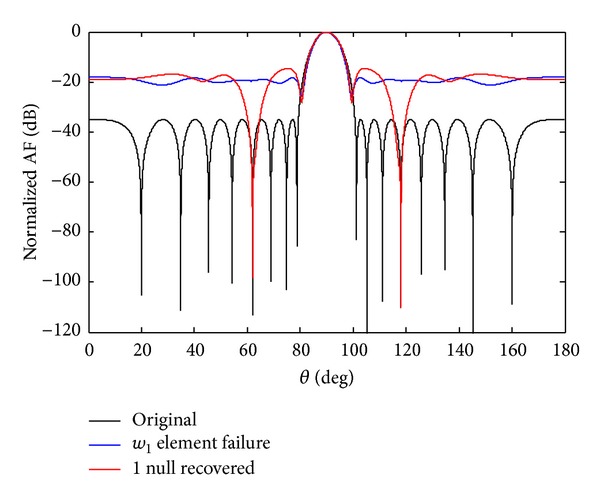
The original radiation pattern, the *w*_1_ sensor failure, and recovery of one null.

**Figure 15:**
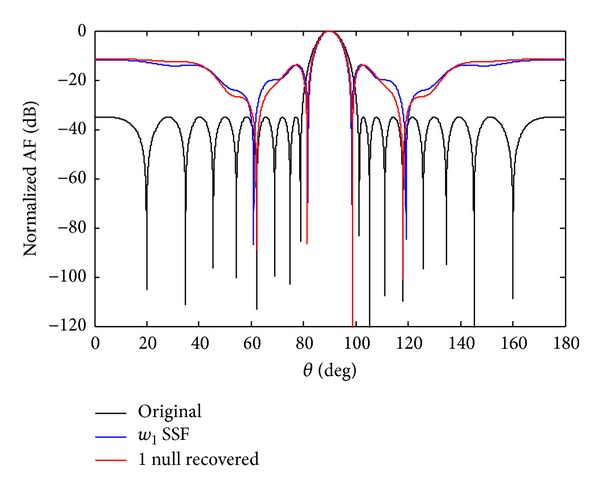
The original radiation pattern, the *w*_1_ SSF, and recovery of one null.

**Figure 16:**
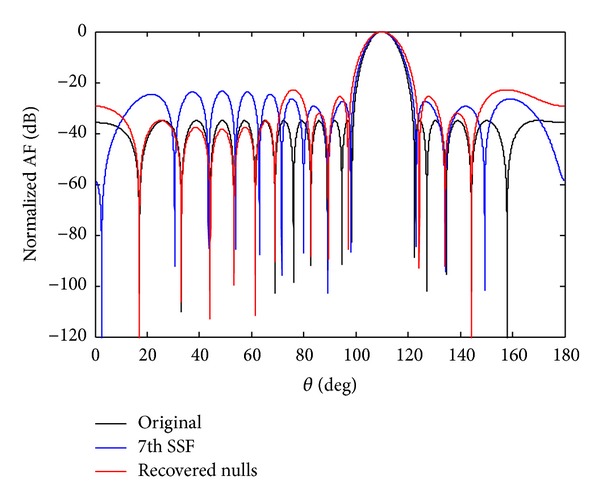
The corrected pattern with main beam pointing at 110° with recovered nulls.

**Table 1:** Parameter used in CADE.

CADE	
Parameters	Setting
Population size	300
Number of generation	500
Value of *F*	0.5
Value of CR	0.5 ≤ CR ≤ 1

**Table 2:** Comparison of FNDL and SLL of the damaged pattern.

Comparison of FNDL and SLL of damage pattern of 7th sensor and SSF			
7th sensor failure		SSF	
FNDL (dB)	SLL (dB)	FNDL (dB)	SLL (dB)
−32.32	−29.45	−88.98	−26.53

**Table 3:** Recovery of one null.

Comparison of NDL and SLL of one sensor failure and SSF				
Correction of 7th sensor failure		Correction of SSF		Recovery of nulls
NDL (dB)	SLL (dB)	NDL (dB)	SLL (dB)	
−104.7	−32.99	−111.5	−28.35	1st null recovered.

**Table 4:** Recovery of two nulls.

Comparison of NDL and SLL of 7th sensor failure and SSF				
Correction of 7th sensor failure		Correction of SSF		Recovery of nulls
NDL (dB)	SLL (dB)	NDL (dB)	SLL (dB)	
−95.3	−29.87	−115.1	−31.12	1st null recovered.
−93.65	−30.75	−101.4	27.07	2nd null recovered.

**Table 5:** Recovery of three nulls.

Comparison of NDL and SLL of 7th sensor failure and SSF				
Correction of 7th sensor failure		Correction of SSF		Recovery of nulls
NDL (dB)	SLL (dB)	NDL (dB)	SLL (dB)	
−111.6	−36.32	−118.5	−34.59	1st null recovered.
−97.57	−34.92	−105.6	−32.68	2nd null recovered.
−95.01	−28.1	−103.3	−24.19	3rd null recovered.

**Table 6:** Recovery of five nulls.

Comparison of NDL and SLL of 7th sensor failure and SSF				
Correction of 7th sensor failure		Correction of SSF		Recovery of nulls
NDL (dB)	SLL (dB)	NDL (dB)	SLL (dB)	
−112.7	−37.34	−120	−37.8	1st null recovered.
−98.16	−35.31	−108.4	−34.93	2nd null recovered.
−95.33	−24.9	−105.3	−22.91	3rd null recovered.
−94.6	−36.42	−102.5	−32.27	4th null recovered.
−97.39	−22.69	−99.68	−25.832	5th null recovered.

**Table 7:** Recovery of three nulls.

Comparison of NDL and SLL of 4th sensor failure and SSF				
Correction of 7th sensor failure		Correction of SSF		Recovery of nulls
NDL (dB)	SLL (dB)	NDL (dB)	SLL (dB)	
−87.56	−22.1	−86.24	−22.64	1st null recovered.
−102.7	−23.7	−97.97	−19.65	2nd null recovered.
−88.81	−21.11	−94.01	−20.01	3rd null recovered.

**Table 8:** Recovery of one nulls.

Comparison of NDL and SLL of *w*_1_ sensor failure and SSF				
Correction of *w*_1_ sensor failure		Correction of SSF		Recovery of nulls
NDL (dB)	SLL (dB)	NDL (dB)	SLL (dB)	
−113.3	−20.02	−115.1	−21.1	1st null recovered.
